# Dialing the glenosphere eccentricity posteriorly to optimize range of motion in reverse shoulder arthroplasty

**DOI:** 10.1016/j.jseint.2024.09.003

**Published:** 2024-09-18

**Authors:** Stefan Bauer, Arnaud Meylan, Jaad Mahlouly, Wei Shao, William G. Blakeney

**Affiliations:** a Centre de l'Épaule et du Membre Supérieur, Ensemple Hospitalier de la Côte, Morges, Switzerland; b School of Surgery, University of Western Australia, Perth, Australia; c Orthopaedic Department, Royal Perth Hospital, Perth, Australia

**Keywords:** Glenosphere, Eccentricity, Reverse shoulder arthroplasty, Range of motion, Impingement, Notching

## Abstract

**Background:**

Friction is the primary cause of notching in reverse shoulder arthroplasty during internal, external rotation (IR/ER), and extension (EXT). To address notching, glenosphere eccentricity (ECC) was introduced. The primary objective of this study was to investigate different positions of glenosphere ECC to determine whether there is an optimal position for impingement-free range of motion.

**Methods:**

In this computer model study, 10 female CTs were analyzed and EXT, ER, IR, and adduction simulated (18 models.) A 135° stem with a +10° liner (145°) was combined with a 25-mm standard/or +3-mm lateralized baseplate (BP) and a 36-mm +2-mm eccentric glenosphere in 4 ECC positions (50° posterior; 30° posterior; 30° anterior; 50° anterior) from the reference position 0° neutral (10 models). Additionally, a concentric 39-mm glenosphere was tested (+2 models). A 0° insert, 135° neck shaft angle (NSA), was tested with a 25-mm standard/or +3-mm BP for 3 configurations (30° posterior; neutral; 39 mm; +6 models).

**Results:**

Compared to the 0° neutral reference position, 30° posterior dialing improved the mean ER (group 145°: 40° vs. 38°, group 145° + 3: 51° vs. 49°, both *P* < .0001), and EXT (group 145°: 35° vs. 34°, *P* = .029, group 145° + 3: 57° vs. 47*°*, *P* = .046, but at the expense of IR (group 145°: 83° vs. 87°, group 145° + 3: 87° vs. 91°, both *P* < .0001). The position 30° anterior increased IR (group 145°: 90° vs. 87°, group 145° + 3: 94° vs. 91°, both *P* < .0001) at the expense of ER (group 145°: 33° vs. 38°, group 145° + 3: 44° vs. 49°, both *P* < .0001) and EXT (group 145°: 24° vs. 34°, *P* = .055, group 145° +3 mm: 39° vs. 47°, *P* = .0042). For group 145°, 0° neutral was the best position for combined EXT + IR (121°) compared to 30° posterior/30° anterior/39 mm/50° posterior/50° anterior (118°/113°/118°/113°/110°, *P* < .0001/*P* = .15/*P* = .076/*P* < .0001/*P* = .074, respectively) and IR + ER (125° vs. 122°/123°/123°/118°/119°/, *P* < .001/*P* = .0028/*P* = .7/*P* < .0001/*P* = .0001, respectively). Lateralization, but most effectively a 135° NSA improved combined EXT + IR + ER + adduction (group 145°: 179° vs. group 135°: 243°, group 145° + 3: 215° vs. group 135° + 3: 276°, *P* = .0019/*P* = .00019, respectively). The influence of position 0°neutral or 30°posterior became marginal with a 135°NSA.

**Conclusion:**

Posterior dialing of the ECC increases EXT and ER but at the expense of IR. Lateralization, but most effectively a 135° NSA, increase impingement-free motion. A larger noneccentric glenosphere on the same BP is a safe all-round solution to prevent ECC positioning outliers.

Glenosphere eccentricity (ECC) was originally introduced to reduce notching in reverse shoulder arthroplasty. In the early designs of reverse shoulder arthroplasty notching occurred in up to 94% of patients postoperatively. Notching is associated with worse patient outcomes^17^^,^^16^ and glenoid loosening.^18^ Notching was initially thought to be a result of impingement of the prosthesis on the scapula in adduction (ADD).^12^ Subsequent research has shown that friction-type impingement in internal (IR), external rotation (ER), and extension (EXT) is the likely culprit.^4^^,^^11^

In our recent study, it was demonstrated that increasing the offset of the posterior scapular pillar from the glenosphere, was associated with increased EXT and ER.^3^ Increasing the offset of the anterior scapular pillar from the glenosphere increased IR. Both were associated with increased ADD. The study also demonstrated that the posterior scapula neck offset from the glenosphere was always smaller than the anterior offset. This is a result of the anatomy of the scapula neck, which has a prominent posterior inferior glenoid tubercle.^14^

The question then arises, whether there is an optimal position to dial the ECC of the glenosphere to enhance range of motion (ROM). We would expect that dialing the ECC posteriorly, might improve ER and EXT, but might this be at the expense of IR? The other possible solution that has been used, is increasing offset in all directions by increasing the glenosphere size.^9^

To analyze the effects of different glenosphere ECC positions on ROM, we conducted a study using computer modeling to simulate various positions and detect impingement-free ROM. We focused on dialing the ECC posteriorly and anteriorly from a commonly used direct inferior, 0° neutral reference position. Additionally, we examined the effects of lateralization and lowering the neck shaft angle (NSA) from 145° to 135°.

## Methods

We analyzed 10 deidentified CT scans of female patients (mean age: 73.9 years, range: 63-83 years; mean height: 161.3 cm, range: 151 cm-172 cm) to create comparative computer models. The retrospective analysis of CT scans was approved by the local ethics committee (CER-VD 020-0049) and patients gave informed consent for the utilization of their deidentified data. Patients presenting with massive rotator cuff tears without joint space narrowing and Sirveaux type E0 glenoids were included. Exclusion criteria consisted of any glenoid erosion or a rotator cuff arthropathy of Hamada type 3 or higher.

Glenosys research and development software (version 10.6.4; Imascap, Brest, France) was used to upload and analyze the digital imaging and communications in medicine data. Automated segmentation was performed by the software before automatically calculating the glenoid version and inclination as previously described.^19^ A neutral reference scapular plane was automatically computed based on 3D reconstruction of all points of the scapula body. The reference points, axis, planes and automated measurement process previously been validated and published.^6^ The best fit native humeral head cut diameter was automatically calculated by a software algorithm and manually corrected by 2 experienced shoulder surgeons until agreement on the sizing was reached. The mean head size was 43.3 mm (range: 41 mm-48 mm).

**Figure 1:**
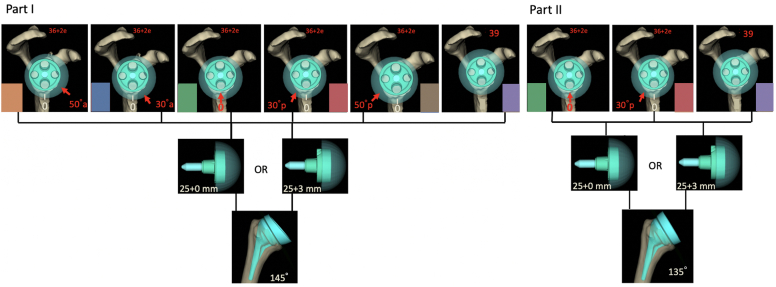
Part I of the study combines a 145° NSA with 2 different baseplates (25-mm standard and +3-mm lateralized), 5 different glenosphere eccentricity positions and a 39-mm noneccentric glenosphere. Part II combines a 135° NSA with 2 different baseplates (25-mm standard and +3-mm lateralized) 2 different glenosphere eccentricity positions and a 39-mm noneccentric glenosphere. *NSA*, neck shaft angle.

Eighteen different computer models were created for each patient CT scan before virtually simulating glenohumeral rigid body motion. EXT, ER, IR, and ADD were recorded. The models consisted of a standardized 135° stem (Perform; Stryker, Kalamazoo, MI, USA) in anatomic retrotorsion with a symmetric 0° or asymmetric 10° insert to end up with a 135° or 145° NSA, a standard or +3-mm lateralized (3lat) 25-mm baseplate (BP) (Fig. 1), and a 36-mm +2-mm eccentric (ecc) glenosphere (Perform; Stryker, Kalamazoo, MI, USA). All BPs were positioned flush with the inferior glenoid margin and in the same orientation with 0° of version, 0° of inclination and at 0 mm of medialization as calculated by the software. The reference position of the glenosphere ECC was neutral, directly inferior. From this position, the glenosphere ECC was dialed 30° and 50° posteriorly and 30° and 50° anteriorly (Fig. 1 and Table I). A 39-mm concentric glenosphere allowing for a circumferential overhang was also used for comparison to the different ECC positions.

**Table I:** Eighteen different models with variable NSA (145° and 135°), baseplate lateralization (25 mm and 25 +3 mm), and position of the glenosphere eccentricity.

BP 25 mm or 25 + 3 mm	Position of the glenosphere eccentricity (36 + 2 mm)
Part I	
145°NSA BP 25 mm (1)	50° posterior
145°NSA BP 25 mm (2)	30° posterior
145°NSA BP 25 mm (3)	Neutral
145°NSA BP 25 mm (4)	30° anterior
145°NSA BP 25 mm (5)	50° anterior
145°NSA BP 25 mm (6)	39-mm GS
145°NSA BP 25 mm + 3 mm (7)	50° posterior
145°NSA BP 25 mm + 3 mm (8)	30° posterior
145°NSA BP 25 mm + 3 mm (9)	Neutral
145°NSA BP 25 mm + 3 mm (10)	30° anterior
145°NSA BP 25 mm + 3 mm (11)	50° anterior
145°NSA BP 25 mm + 3 mm (12)	39-mm GS
Part II	
135°NSA BP 25 mm (13)	30° posterior
135°NSA 25 mm (14)	Neutral
135°NSA 25 mm (15)	39-mm GS
135°NSA 25 mm + 3 mm (16)	30° posterior
135°NSA 25 mm + 3 mm (17)	Neutral
135°NSA 25 mm + 3 mm (18)	39-mm GS

*BP*, baseplate; *NSA*, neck shaft angle; *GS*, glenosphere.

In part I of the study (Table I and Fig. 1), a 145° NSA stem + insert configuration was used with a standard BP^2^^,^^3^^,^^4^^,^^5^^,^^6^^,^^7^ and a +3-mm lateralized BP^8^^,^^8^^,^^9^^,^^10^^,^^11^^,^^12^ and all configurations were tested to demonstrate the effect of changing the position of glenosphere ECC (red arrow in Fig. 1). In part II (Table I and Fig. 1), a 135° NSA was used with and without BP lateralization^13^^,^^14^^,^^15^^,^^17^^,^^16^^,^^18^.

### Primary and secondary outcome variables

EXT, ER, IR, and 2 combined motion values (EXT + IR and ER + IR) were the primary outcome variables in combination with a 145° NSA and different BP lateralizations. ADD and the combined motion “EXT + IR + ER + ADD” for the comparison of different BP lateralizations and NSAs were secondary outcome variables.

### Statistical analysis

R language was used for statistical analyses and illustrations (version 3.4.1; R Foundation for Statistical Computing, Vienna, Austria). Statistical significance was assumed with 2-tailed *P* < .05. For internal group comparison, paired Student T-test was performed for comparison between each model within groups for each variable. For between-group comparison, unpaired Student T-test was performed for comparison between each group for each variable. Cohen’s d was calculated for measuring effect size. Sawilowsky’s descriptors for magnitude of d (0.01-2.0) can be applied with d = 0.01 (very small), 0.20 (small), 0.50 (medium), 0.80 (large), 1.20 (very large), and 2.0 (huge).

## Results

**Figure 2:**
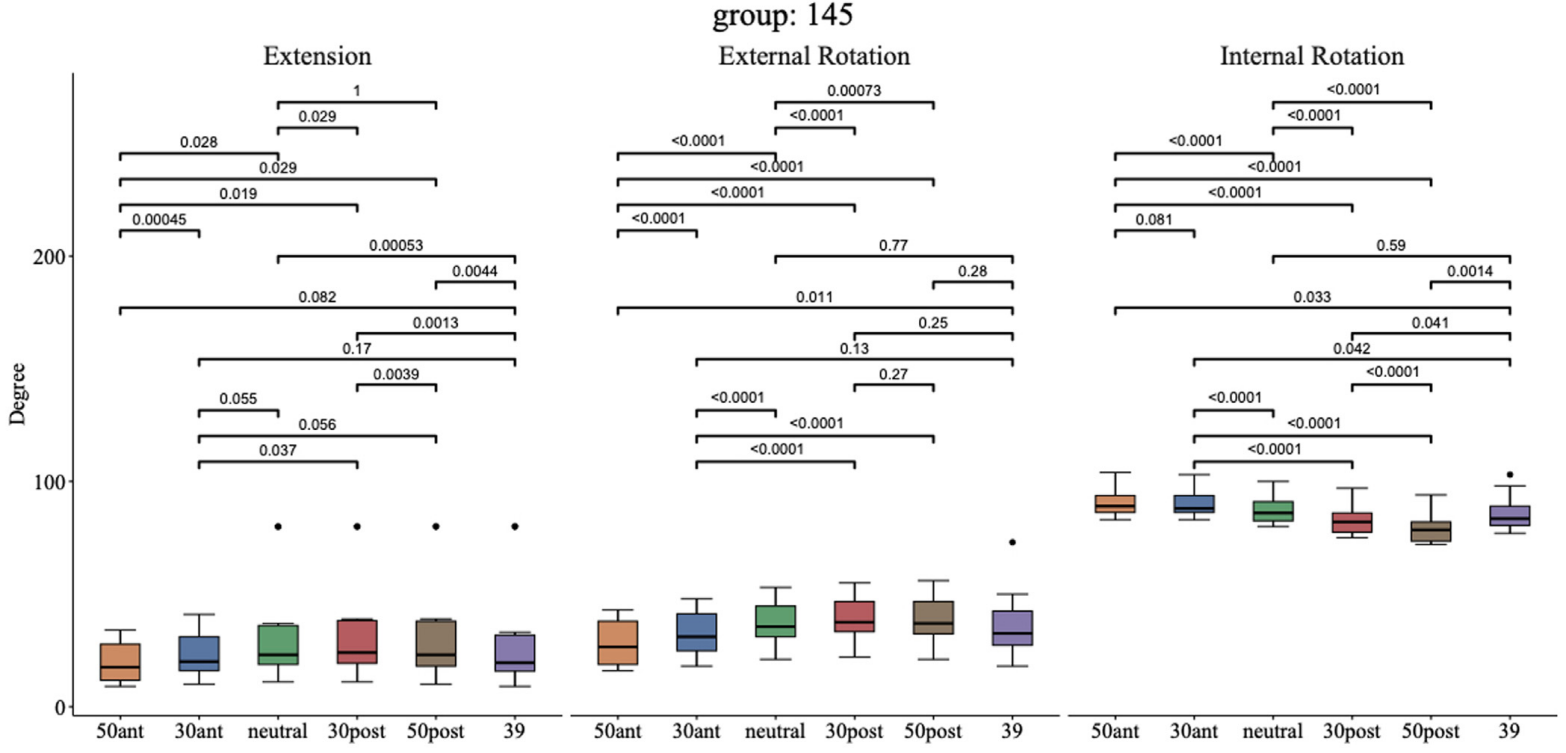
Extension, external rotation, and internal rotation for different glenosphere eccentricity positions combined with a 145° NSA without baseplate lateralization (models 1-6). *NSA*, neck shaft angle.

As illustrated in Fig. 2, for group 145° without BP lateralization, the ECC position 30° posterior showed better mean EXT (35° [95% CI: 17°-53°] vs. 34° [95% CI: 16°-52°], *P* = .029, d = 0.82) and better mean ER (40° [95% CI: 32°-47°] vs. 38° [95% CI: 30°-45°], *P* < .0001, d = 3.48) than the 0° neutral reference position. A 39-mm glenosphere showed decreased mean EXT compared to 0°neutral (34° [95% CI: 16°-52°] vs. 32° [95% CI: 13°-50°], = 0.00053, d = 1.66) and no significant difference for the mean ER (38° [95% CI: 30°-45°] vs. 37° [95% CI: 25°-48°], *P* = .77, d = 0.10) and mean IR (87° [95% CI: 83°-92°] vs. 86° [95% CI: 80°-92°], *P* = .59, d = 0.18) compared to 0°neutral. There was an increased mean IR for 0° neutral compared to 30° posterior (87° [95% CI: 83°-92°] vs. 83° [95% CI: 78°-87°], *P* < .0001, d = 6.29) and for 30° anterior compared to 0° neutral (90° [95% CI: 85°-94°] vs. 87° [95% CI: 83°-92°], *P* < .0001, d = 4.43).

**Figure 3:**
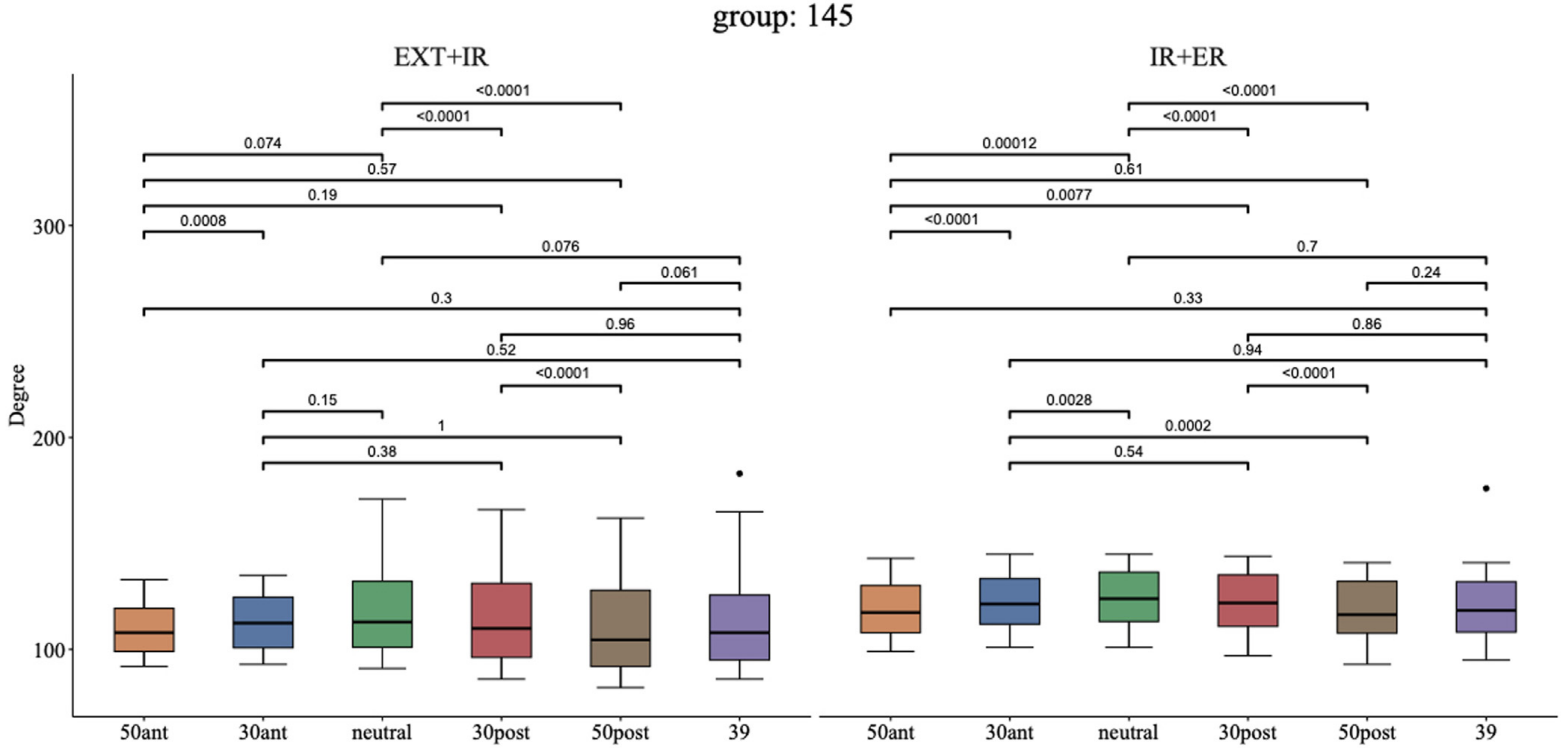
Combined extension + internal rotation and internal rotation + external rotation for different glenosphere eccentricity positions combined with a 145° NSA without baseplate lateralization (models 1-6). *NSA*, neck shaft angle; *EXT*, extension; *IR*, internal rotation; *ER*, external rotation.

As shown in Fig. 3, 0°neutral was best for the mean EXT + IR and better than 30° posterior (121° [95% CI: 101°-142°] vs. 118° [95% CI: 98°-138°], *P* < .0001, d = 2.21). Combining the mean IR + ER, the best compromise was seen for 0° neutral which showed better combined motion than 30° anterior (125° [95% CI: 114°-135°] vs. 123° [95% CI: 112°-133°], *P* = .0028, d = 1.29) and 30° posterior (125° [95% CI: 114°-135°] vs. 122° [95% CI: 111°-134°], *P* < .0001, d = 2.13). For EXT + IR, the ECC outlier positions 50° anterior and 50° posterior underperformed compared to 0° neutral (110° [95% CI: 100°-120°], *P* = .073, d = 0.64; 113° [95% CI: 93°-134°], *P* < .0001, d = 3.89, respectively) and the same was seen for IR + ER (119° [95% CI: 108°-129°], *P* = .0001, d = 2.04; 118° [95% CI: 107°-130°], *P* < .0001, d = 3.83, respectively).

**Figure 4:**
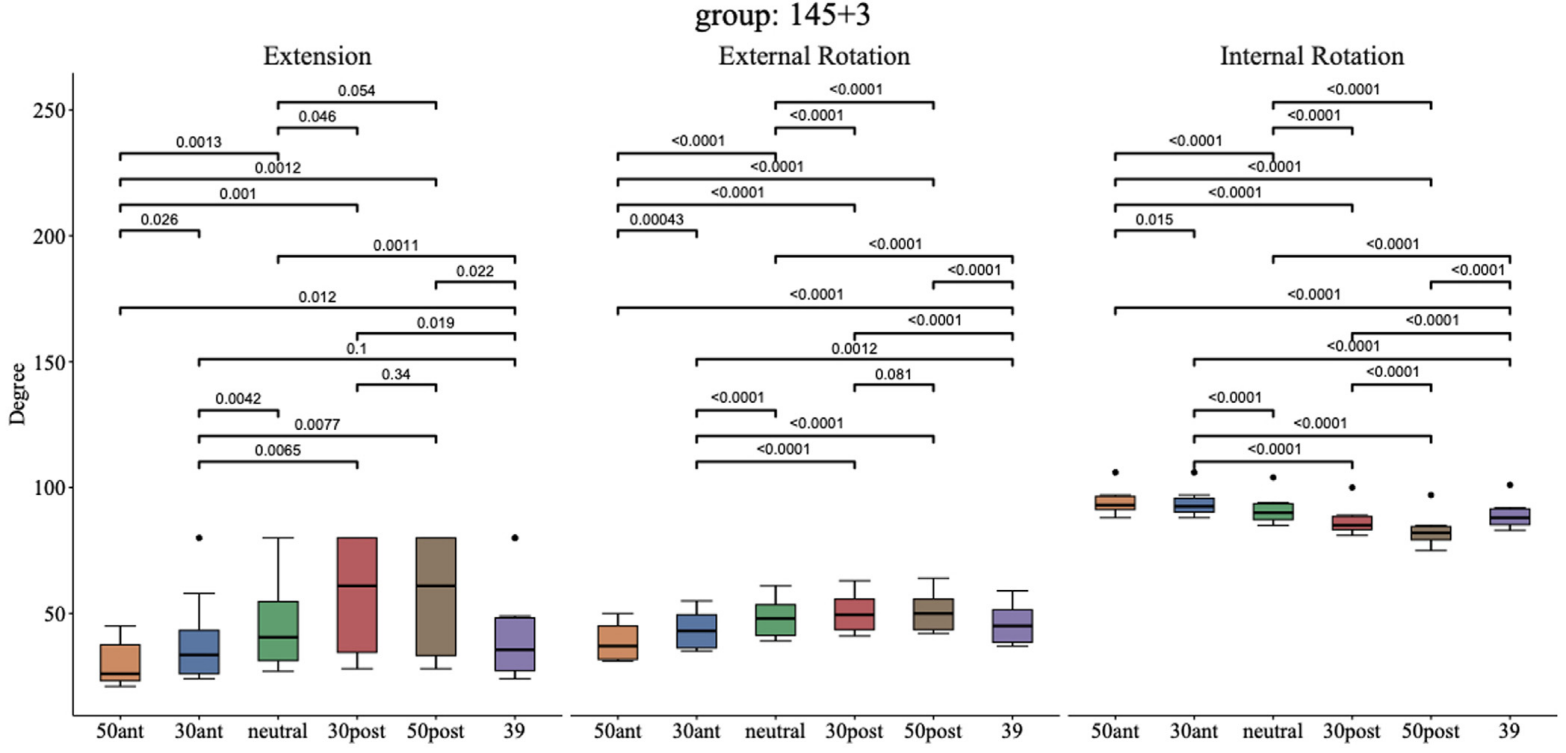
Extension, external rotation, and internal rotation for different glenosphere eccentricity positions combined with a 145° NSA with +3-mm baseplate lateralization (models 7-12). *NSA*, neck shaft angle.

Fig. 4 shows group 145°+3 mm. For 30° posterior compared to 0° neutral, there was an increase in the mean EXT (57° [95% CI: 40°-75°] vs. 47° [95% CI: 33°-61°], *P* = .046, d = 0.73) and mean ER (51° [95% CI: 45°-57°] vs. 49° [95% CI: 43°-54°], *P* < .0001, d = 3.48) in favor of 30° posterior. There was a highly significant increase of the mean IR for 0° neutral compared to 30° posterior (91° [95% CI: 87°-95°] vs. 87° [95% CI: 83°-90°], *P* < .0001, d = 5.22) and for 30° anterior compared to 0° neutral (94° [95% CI: 90°-97°] vs. 91° [95% CI: 87°-95°], *P* < .0001, d = 5.59).

**Figure 5:**
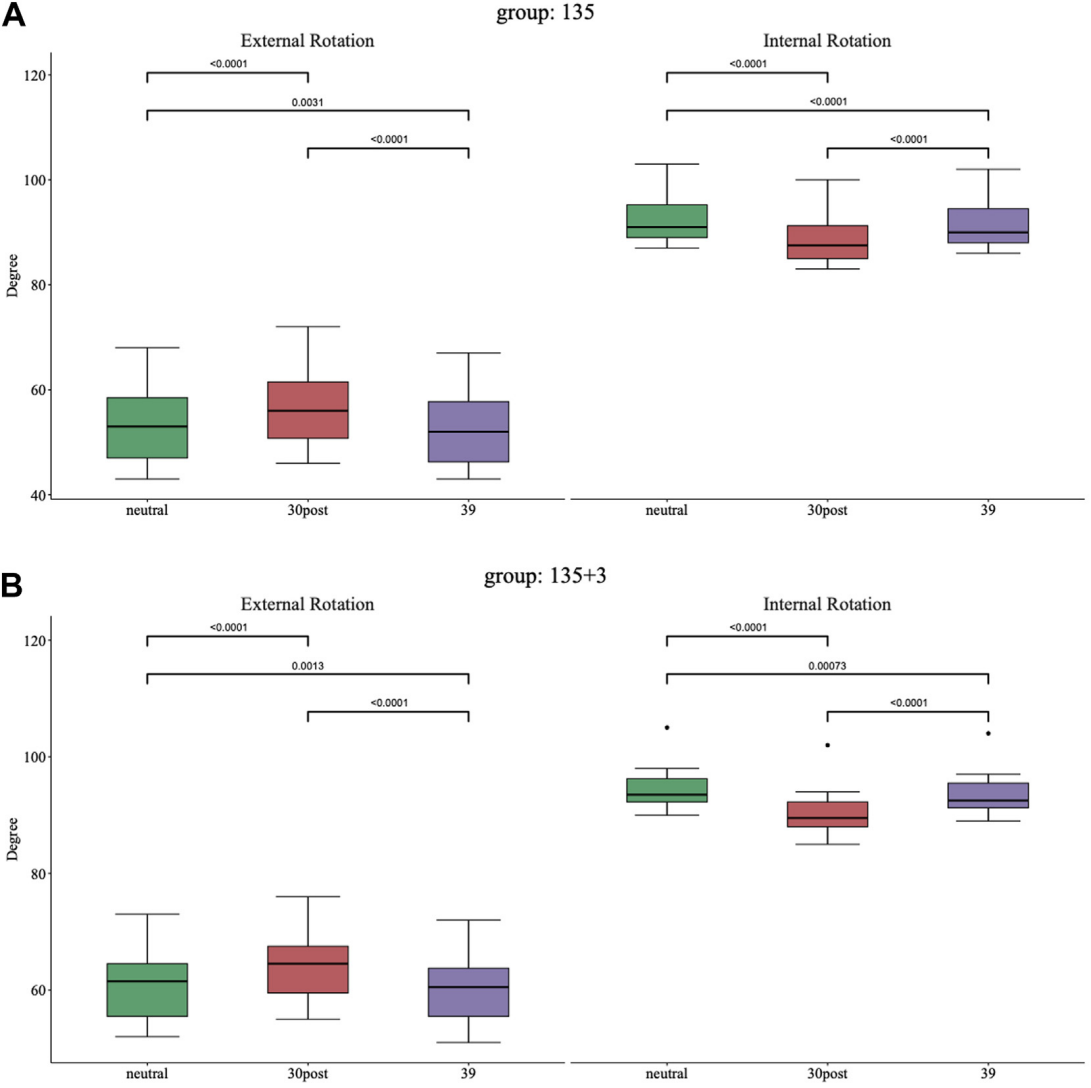
(**A**) External rotation and internal rotation for different glenosphere eccentricity positions combined with a 135° NSA without baseplate lateralization (models 13-15). (**B**) External rotation and internal rotation, 135°NSA with +3-mm baseplate lateralization (models 16-18). *NSA*, neck shaft angle.

Fig. 5*A* illustrates the group 135° without BP lateralization. For the mean ER, 30° posterior was better than 0° neutral (57° [95% CI: 51°-63°] vs. 54° [95% CI: 48°-60°], *P* < .0001, d = 6.83), and 0° neutral was better than a 39-mm glenosphere (54° [95% CI: 48°-60°] vs. 53° [95% CI: 47°-59°], *P* < .0031, d = 1.26) (*P* < .0031). For the mean IR, 0° neutral outperformed 30° posterior (92° [95% CI: 89°-96°] vs. 88° [95% CI: 85°-92°], *P* < .0001, d = 6.87) and a 39-mm glenosphere (91° [95% CI: 88°-95°], *P* < .0001, d = 2.85).

Fig. 5*B* shows the group 135°+3-mm BP lateralization with similar relationships between the ECC positions but increased total EXT and IR.

**Figure 6:**
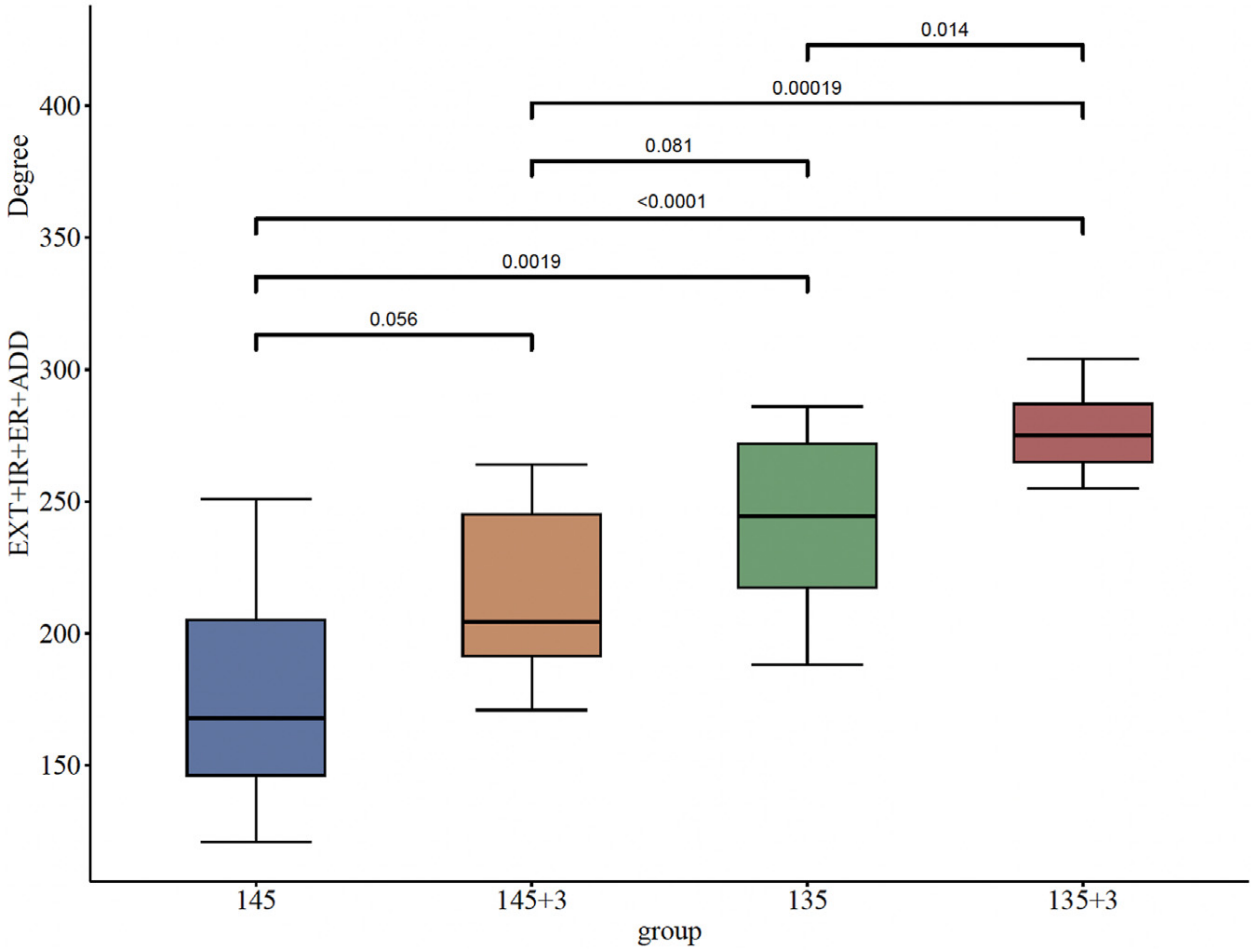
Comparison of combined extension + internal rotation + external rotation + adduction (0° neutral position) for a 145° NSA and 135° NSA with and without baseplate lateralization (models 1-18). Order of increasing means. *NSA*, neck shaft angle; *EXT*, extension; *IR*, internal rotation; *ER*, external rotation; *ADD*, adduction.

Combined EXT + IR + ER + ADD was used to represent total notching relevant ROM as displayed in Fig. 6. It was calculated for the 0° neutral ECC position. There was an increased ROM for +3-mm lateralization. Lowering the NSA from group 145° to group 135° and from group 145°+3 mm to group 135°+3 mm most effectively increased notching relevant combined ROM (179° [95% CI: 148°-210°] vs. 243° [95% CI: 218°-267°], *P* = .0019, d = 1.64; 215° [95% CI: 190°-239°] vs. 276° [95% CI: 266°-287°], *P* = .00019, d = 2.34, respectively).

All statistical comparisons, both within groups (intragroup) and between groups (intergroup), are available in the supplementary tables (Supplementary files 1 and 2).

## Discussion

The key finding of this study is that dialing the ECC of the glenosphere has a significant effect on the ROM. As was hypothesized, posterior offset increased EXT and ER, but at the expense of IR. Anterior ECC increased IR, but at the expense of ER and EXT.

The benefits and risks of glenoid vs. humeral-sided and combined lateralization have been discussed recently.^2^ Using a lateralized base plate and changing the NSA affected both, the ROM and also the optimal position of the glenosphere ECC. Using a 145° NSA stem with no BP lateralization, placing the ECC directly inferior was the best position to maximize total ROM, as IR was too reduced by placement of the BP posteriorly. When the BP was lateralized by 3 mm, maximal ROM was achieved with the BP dialed 30° posterior. Lateralization by 3 mm in itself increased ROM in all positions. The use of a 135° NSA with and without BP lateralization was the most powerful factor for significantly increased motion, with marginal differences achieved between neutral or posterior positioning of the glenosphere.

Inferior glenosphere offset has been shown to be associated with increased ROM in a clinical study of 160 patients by Haidamous et al.^8^ This was achieved by using an eccentric glenosphere, increasing the glenosphere size, and ensuring inferior positioning of the base plate. By moving the humerus away from the scapula pillar, computer modeling studies have demonstrated an improved ROM. A recent study by our group demonstrated the importance of the posteroinferior distance from the scapular margin to the glenosphere, termed posteroinferior relevant scapular neck offset, for EXT, ER, and ADD.^3^ The anteroinferior distance, termed anteroinferior relevant scapular neck offset is important for IR and ADD. Lateralization, glenoid ECC, and glenosphere size will also increase the relevant scapular neck offset and the combination of all these methods has the greatest effect. The posteroinferior relevant scapular neck offset was always significantly smaller than the anteroinferior relevant scapular neck offset. In keeping with this observation, in the present study, posterior dialing of the glenosphere ECC was shown to be valuable in maximizing EXT and ER.

Decreasing the NSA from 145° to 135° has very minimal influence on humeral lateralization.^20^ Its main effect is to tilt the polyethylene insert away from the scapular pillar, and in so-doing increase impingement free ROM. The risk of using a 135° is of introducing instability.^7^^,^^15^

In the present study we have looked at the combination of IR and EXT in the different prosthesis combinations. Gerber et al demonstrated the importance of functional EXT for IR to the back.^10^ They found that at least 40° of active EXT is necessary to achieve a functional IR, allowing placement of the hand behind the back to reach the lumbar spine and higher.

Interestingly, in this study a larger 39-mm glenosphere size was not shown to improve ROM beyond an eccentric 36-mm glenosphere. This is likely because the glenosphere offset in the important inferior direction is similar between the 2 combinations. With the +2-mm eccentric 36-mm glenosphere it is 7.5 mm, compared with 7 mm for the 39-mm glenosphere. With the 39-mm concentric glenosphere, the offset is the same in all directions, hence removing the importance of dialing to the best position. The Australian National Joint Registry reports lower rates of revision in females with 38-40 mm and in males with > 40 mm glenospheres compared to sizes < 38 mm.^1^ A larger glenosphere may be a more forgiving all-round solution to prevent underperforming outlier ECC positions such as too anterior creating early posterior impingement and too posterior creating anterior impingement in this study.

This study has some limitations. Glenosphere ECC, when associated with certain implant configurations, for example, an inferiorly placed, small BP combined with a larger eccentric glenosphere, may lead to excessive inferior overhang and humeral distalization with the potential to jeopardize the soft tissues and the axillary nerve.^5^^,^^13^ However, distalization and overhang with eccentric glenospheres and size-matched BPs like in our computer model study have been clinically shown to be safe.^8^ This study is a computer modeling study and is unable to consider scapulothoracic motion or the influence of the soft tissues and muscle forces.

Although the statistical effect size d was at least large (Cohen’s d > 0.80) for significant differences in this study, it is arguable whether some of the smaller changes in ROM have a noticeable clinical effect on patients. However, in patients with low body mass index, notching has been shown to be increased,^16^ soft tissue impingement may be reduced and maximal impingement-free glenohumeral ROM is advantageous to prevent notching. It is useful for the surgeon to understand the effects of implant position changes on postoperative ROM. The positions of the glenosphere ECC were chosen to demonstrate the direction of the effect of these changes on ROM. In clinical practice, it may be difficult or impossible to place the ECC of the glenoid in the more extreme positions. Certainly, when performing a delto-pectoral approach, it is more difficult to place the ECC posterior because of impingement with the humeral retractor. The surgeon needs to be cognizant of this and understand the effect of placing the ECC anteriorly, which is technically easier.

## Conclusion

The results of this study show that dialing the glenosphere ECC posteriorly optimizes impingement-free EXT and ER but at the expense of IR. Lateralization and most importantly a lower NSA of 135° enhance the ROM to prevent impingement. A larger noneccentric glenosphere on the same BP is a safe all-round solution to prevent ECC positioning outliers creating early anterior or posterior impingement as seen with the 50° posterior and 50° anterior positions in this study.

## Acknowledgments

The authors express thanks to the Australian government for the support of this work as part of a Research Training Program PhD scholarship kindly granted for the main author (Stefan Bauer).

## Disclaimers:

Funding: The Australian government provided support for this work as part of a Research Training Programm PhD scholarship kindly granted for the main author (Stefan Bauer).

Conflicts of interest: The authors, their immediate families, and any research foundations with which they are affiliated have not received any financial payments or other benefits from any commercial entity related to the subject of this article.

## Appendix A Supplementary data

20240807 paired_ttest_output

20240807_group_ttest_output
